# Evaluation of quantitative muscle MRI and an intelligent phenotyping housing system as advanced phenotyping methods in a mouse model of calpain 3‐deficient muscular dystrophy

**DOI:** 10.1002/ame2.70193

**Published:** 2026-04-06

**Authors:** Nicolina Südkamp, Marlena Rohm, Gabriele Russo, Xavier Helluy, Abdulhadi Kocabas, Martijn Froeling, Felix Kleefeld, Denise Manahan‐Vaughan, Tobias Ruck, Frank Jacobsen, Matthias Vorgerd, Johannes Forsting, Lara Schlaffke

**Affiliations:** ^1^ Department of Neurology BG‐University Hospital Bergmannsheil Bochum, Ruhr University Bochum Bochum Germany; ^2^ Heimer Institute for Muscle Research BG‐University Hospital Bergmannsheil Bochum Bochum Germany; ^3^ Department of Neurophysiology, Medical Faculty Ruhr University Bochum Bochum Germany; ^4^ Department of Biopsychology, Faculty of Psychology, Institute of Cognitive Neuroscience Ruhr University Bochum Bochum Germany; ^5^ Department of Radiology University Medical Centre Utrecht Utrecht The Netherlands; ^6^ Department of Information Technology FH Dortmund, University of Applied Sciences and Arts Dortmund Germany; ^7^ University Hospital of Pediatrics and Adolescent Medicine Bochum Ruhr‐University Bochum Bochum Germany

**Keywords:** calpainopathy, fully automated home‐cage monitoring system, motor function, mouse model, qMRI

## Abstract

Calpainopathy is a rare genetic myopathy without causal treatment available. Recent advances have produced promising treatment strategies, including genetic treatment and immunomodulation, that are currently being tested pre‐clinically in murine models. Traditional behavioral assays frequently fail to detect early motor deficits in corresponding mouse models. Thus, this study investigated whether more sophisticated measures, such as quantitative magnetic resonance imaging (qMRI) and automated motor assessments can reveal early pathological changes in a mouse model of calpainopathy, particularly in the subclinical stages. In this study, *Calpain 3*‐deficient mice (Capn3‐deficient) and wild‐type controls underwent a standardized qMRI protocol on a 7T scanner at 5 and 15 months of age. Fat fraction (FF), water T2 relaxation time (wT2), and diffusion metrics were analyzed across selected hindlimb muscles. Additionally, voluntary motor activity was monitored using an intelligent phenotyping housing system. Motor activity assays revealed minimal and inconsistent genotype effects. Sex significantly affected qMRI measures, altering fat distribution and wT2 at 5 and 15 months. At 5 months, no significant differences were detected between genotypes. At 15 months, Capn3‐deficient mice exhibited elevated wT2 in the soleus and gastrocnemius muscles, correlating with endomysial immune cell infiltration. Diffusion parameters and FF remained unchanged. Taken together, the results show that muscle qMRI, particularly T2 mapping, can sensitively detect subclinical muscle changes in aging Capn3‐deficient mice, pointing towards inflammatory processes. Pronounced sex differences and measurement variability underscore the need for larger, sex‐balanced cohorts. Extending follow‐up beyond 15 months will likely increase sensitivity for later‐stage pathology.

## Graphical abstract

We applied quantitative MRI of the lower limb and automated home‐cage phenotyping to a mouse model of calpainopathy to detect early disease changes. At 15 months, calpain 3‐deficient mice showed increased water T2 values correlating with immune cell infiltration in the soleus and gastrocnemius muscles, while assessment of motor activity revealed only subtle, sex‐specific differences. These findings suggest qMRI as a sensitive non‐invasive tool for detecting early pathology, highlighting the importance of considering sex differences when muscle composition and function are investigated in mouse models.

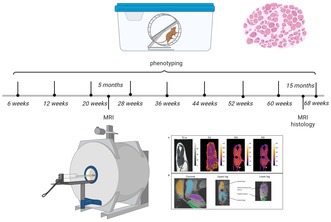

## 1 INTRODUCTION

Calpainopathy, or Limb Girdle Muscular Dystrophy (LGMD) 2A/R1, is an autosomal recessively inherited disease, which is caused by mutations in the gene coding for calpain 3 protein (CAPN3), a muscle specific protease.^1^, ^2^ CAPN3 plays a role in the regulation of apoptosis, the regulation of sarcomere structure, and the differentiation of myocytes.^1^, ^3^, ^4^ Clinically, patients exhibit a progressive atrophy and weakness of the proximal limb girdle muscles, which leads to a loss of walking ability within the first decades of life.^5^, ^6^ Although no treatment is available, recently, novel treatment options, such as gene therapy and immunomodulation, have been developed and are currently being evaluated in murine preclinical studies.^7^, ^8^ Because clinical outcome measures often miss early muscle involvement and disease progression, and muscle biopsy is invasive and therefore impractical, developing sensitive noninvasive biomarkers is essential.^9^ This applies not only to patients^10^ but also to mouse models, given that most potential therapeutics are still tested in murine disease models.^8^ In mouse models, standard motor function assessments, such as grip strength, wire hanging test and beam walk test, are variable and prone to confounders and therefore not sensitive enough to detect early restrictions of motor function.

In recent years, quantitative magnetic resonance imaging (qMRI), including comprehensive measures of water T2 relaxation time, Dixon‐based imaging and diffusion tensor imaging, has emerged as a promising option for monitoring disease progression in different neuromuscular diseases.^9^, ^11^, ^12^ While Dixon‐based imaging is used to calculate fat‐fraction (FF) in muscle, measures of water T2 relaxation time (wT2) reflect active muscle damage such as inflammation or myoedema.^13^ Additionally, diffusion tensor imaging (DTI) can detect early macro‐ and microstructural changes.^14^, ^15^ Furthermore, recent studies have also revealed alignment of qMRI parameters with histopathological findings.^16^, ^17^ In calpainopathy, qMRI studies have emphasized characteristic patterns of muscle involvement with a predominant muscle‐fat replacement of hamstrings, soleus and gastrocnemius medialis muscles that correlates to clinical impairment.^10^, ^18^, ^19^ Longitudinal data showed high sensitivity of FF for monitoring disease progression in LGMD 2A/R1 patients,^20^ supporting qMRI metrics as a promising outcome measure.

An equivalent qMRI scanning protocol for rodents that includes Dixon‐based imaging, T2 mapping, and DTI has been adapted for lower limb studies.^21^ In a mouse model of Pompe disease, FF and wT2 were stable over 8 months, whereas the mean diffusivity (MD) and radial diffusivity (RD) correlated with fiber size and autophagic markers, suggesting diffusion MRI reveals autophagic build‐up.^21^ In chronic denervation models, calf qMRI showed wT2 and DTI changes and was proposed as a sensitive surrogate of motor unit integrity in limb musculature of mice.^22^

The currently available calpainopathy mouse model shows minimal restrictions of motor function within 15 months despite marked loss of full‐length CAPN3 and characteristic histological features, such as fiber atrophy, fiber size variability, and centralized nuclei.^23^ Therefore, more sensitive methods for in vivo phenotyping for this mouse model are warranted. In this study, we evaluated the efficacy of muscle qMRI combined with an intelligent phenotyping housing system (IPHS). We hypothesized that qMRI and IPHS can detect subclinical muscle pathology in Capn3‐deficient mice prior to overt motor dysfunction.

## 2 METHODS

### 2.1 Animals

In this study, male and female 129S4/SvJaeJ‐*Capn3* *^em5Lutzy^*/J mice were utilized as an animal model for calpainopathy (JAX stock #031211, referred to as Capn3‐deficient), while male and female129S4/SvJaeJ mice (JAX stock #009104) served as controls (referred to as WT). The transgenic animals possess a homozygous 1759 nucleotide deletion in the *Capn3* gene, which encodes the muscle tissue‐specific protease CAPN3, resulting in progressive muscular dystrophy. The mice were housed in custom‐made ventilated and acclimatized holding cabinets within a rodent housing room that operated on a 12‐h light/dark cycle, with access to food and water ad libitum. Experiments were conducted in accordance with the European Communities Council Directive of September 22, 2010 (2010/63/EU) regarding the care of laboratory animals and followed the guidelines of the German Animal Protection Law. The experiments were pre‐approved by the North Rhine‐Westphalia (NRW) State Authority (Landesamt für Verbraucherschutz und Ernährung, NRW).

### 2.2 Intelligent phenotyping housing system (IPHS)

At 4 weeks of age, a radio frequency identification (RFID) chip was implanted in the neck of the mice while they were anesthetized with 3% isoflurane. Two weeks after implantation monitoring started. Every 8 weeks, and until the age of 15 months, Capn3‐deficient and WT mice (t0 = 6 weeks, t1 = 12 weeks, t2 = 20 weeks = 5 months, t3 = 28 weeks, t4 = 36 weeks, t5 = 44 weeks, t6 = 52 weeks, t7 = 60 weeks, t8 = 68 weeks = 15 months; *n* = 4 per genotype and sex, separate rounds for each sex) were housed in an IPHS (IntelliCage®, TSE Systems, Germany) for 7 days at each timepoint. Each first day was excluded from the analysis. Animal movement was constantly monitored and recorded by means of the implanted RFID chip. This allowed for uninterrupted social interactions and environmental exploration. The IPHS contained three running wheels (see Figure S1), to assess motor movement. Activity in running wheels was recorded and analyzed at different timepoints (t0–8) for female (*n* = 4 per genotype until t5, then one WT mouse died of an unknown cause) and male mice (*n* = 4 per genotype) separately, using the IntelliMaze Software 5.1.9 (TSE Systems, Germany). During their use, the duration, speed, and distance covered were automatically recorded for each session. The data were analyzed by calculating the average duration, distance, and average and maximum speed for all sessions of each mouse during one week in the IPHS, with one session defined as a single entry and exit through the gates leading to the running wheels. Additionally, maximum duration and distance of a single run were analyzed. A single run was defined as running activity without interruption.

### 2.3 MRI acquisition protocol

Magnetic resonance imaging (MRI) was performed using a 7 tesla horizontal bore small animal MRI system (70/30 USR, Bruker BioSpec, Germany), consistent with previously established methodologies.^21^ Radiofrequency transmission was facilitated through an 80 mm quadrature birdcage resonator, while signal reception was achieved using a 20 mm single‐loop surface coil. Imaging data were acquired with ParaVision 6 software. Prior to imaging, mice were anesthetized using 3% isoflurane in a 4:1 mixture of air and oxygen for approximately 3 min. A thin layer of D‐Panthenol (Bepanthen eye and nose ointment, Bayer, Germany) was applied to the eyes to maintain corneal hydration during the procedure. Animals were positioned on a custom 3D‐printed heated support platform, specifically designed to align with the surface coil at the level of the lower limbs. The left hindlimb was adjusted to form a 90° angle between the tibia and femur. Anesthesia was maintained at 1.7% isoflurane, and normothermia was maintained throughout the imaging session. The imaging protocol, adapted from human protocols for application in rodents,^21^ had a total duration of approximately 60 min (for imaging protocol see Table S1). The left upper and lower hindlimb regions were scanned independently using diffusion‐weighted imaging (DWI) for diffusion tensor imaging (DTI) assessment, as well as multi‐echo sequences for wT2 relaxation time. Imaging slices were acquired perpendicular to the muscle fiber orientation, beginning approximately 3 mm distal to the knee joint (see Figure S2). DWI was executed using a multi‐shell single‐shot echo planar imaging (EPI) Stejskal–Tanner sequence, while wT2 measurements were obtained via a multi‐slice, multi‐echo (MSME) sequence. Additionally, a 3D multi‐echo gradient echo (MEGE) sequence was employed to capture Dixon‐based fat quantification data across the entire limb. For MSME, the number of echoes was 18 (84.9/4.71 = 18.02), Echo Spacing was 4.718 ms, starting at 4.72 ms and ending at 84.93 ms. For the Dixon method, the number of echoes was 10. Echo Spacing started at 1.905 ms, 1.59 ms spacing, ended at 16.19 ms. The flip angle used was 8°. This flip angle is much smaller than the Ernst angle of muscle fibers (28°) for a spoiled gradient echo (assuming T1_muscle = 1.6 s) in order to reduce saturation of muscle fiber signal and thus strong T1 weighted contrast between fat and muscle fibers. The goal being to have a measurement which reflects proton density alone. Assuming T1_fat 600 ms and T1_fiber 1600 ms, the signal intensity ratio S_fat/S_fiber = 1.046. Gradient duration was 3.0 ms, gradient separation time was 10.04 ms. The maximum b‐value achievable with 100% gradient strength was 1076.26 s/mm^2^.

### 2.4 MRI data processing and analysis

Data analysis protocols were adapted from established human skeletal muscle imaging studies,^24^ using QMRITools (http://www.qmritools.com/) implemented in Mathematica 13.14, following methodologies previously described.^21^ The IDEAL algorithm was used to preprocess Dixon‐based datasets, producing quantitative fat fraction maps alongside reconstructed in‐phase (IP) and out‐of‐phase (OP) images.^25^ wT2 relaxation times were estimated using an extended phase graph (EPG) fitting technique based on a two‐compartment model that differentiates between water and lipid signals.^26^ DWI data were processed to generate DTI metrics. This included denoising using a principal component analysis (PCA)‐based algorithm, followed by corrections for motion artifacts and eddy current‐induced distortions. Tensor fitting was performed by incorporating intravoxel incoherent motion (IVIM) effects and utilizing the iteratively weighted linear least squares (iWLLS) method.^27^, ^28^ From each voxel, three eigenvectors (v1–v3) and their associated eigenvalues (*λ*1–*λ*3) were computed to derive diffusion parameters including fractional anisotropy (FA), mean diffusivity (MD), and radial diffusivity (RD) (illustrated in Figure S2).

Anatomical segmentation of key muscle groups – including the tibialis anterior, gastrocnemius complex (including the soleus), quadriceps, and hamstrings – was performed manually using 3D Slicer software (v4.4, https://www.slicer.org/) based on anatomical references from a standardized mouse atlas. Resulting segmentations were subsequently eroded by one voxel to reduce partial volume effects and were spatially co‐registered to the DTI and T2 image datasets. A few datasets had to be excluded from the analysis due to technical or anatomical issues during the scanning.

### 2.5 Histological staining

After anesthesia with 3% isoflurane, mice were sacrificed by cervical dislocation after the MRI scan at the age of 15 months. Cryosections of 5 μm thickness of gastrocnemius and soleus muscle were prepared, and Hematoxylin and Eosin (H&E) staining was performed according to standard procedures.^29^

### 2.6 Statistical analysis

A three‐way ANOVA was used to assess the effects of genotype, sex, and age on the MRI parameters. The analysis was considered exploratory due to limited statistical power. Before using the ANOVA as statistical test, normality was tested with Shapiro–Wilk test.

Longitudinal comparisons of motor activity used two‐way repeated‐measures ANOVA or a mixed effects model approach with either Tukey's or Šídák's multiple comparisons. All statistics were conducted using Python 3.7 with statsmodel (v0.13.2), statannot (v0.2.3) and scipy (v1.7.3), Microsoft Excel and GraphPad Prism (v10.4.1). Figures were created with GraphPad Prism (v10.4.1). Data are expressed as the mean ± standard deviation (*p < 0.05, **p < 0.01, ***p < 0.001, ****p < 0.0001).

## 3 RESULTS

### 3.1 In vivo phenotyping with IPHS detected slight differences between genotypes until the age of 15 months

During phenotyping with IPHS, no significant differences between Capn3‐deficient and WT mice were observed when duration, distance, maximum, or average speed of runs over the time course were compared per sex. Comparison of single timepoints revealed slight differences between different genotypes, as described below.

**FIGURE 1:**
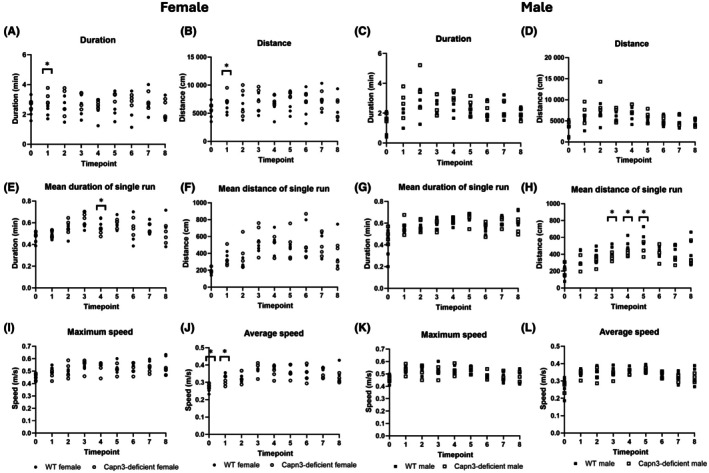
Results of in vivo phenotyping using the IPHS. Female Capn3‐deficient mice showed a higher duration of a session at timepoint 1 compared to WT mice (A). Distance was higher in female Capn3‐deficient mice at timepoint 1 compared to WT mice (B). No differences were observed in male mice regarding the mean duration (C) and distance (D). At timepoint 4, female Capn3‐deficient mice presented a reduced mean duration of a single run compared to WT mice (E). The mean distance of single run did not differ between female Capn3‐deficient and WT mice (F). Male mice did not show differences in the mean duration of single run (G), but male Capn3‐deficient mice presented a shorter distance for single run at timepoint 3, 4 and 5 compared to WT mice of the same age (H). Maximum speed was similar between WT and Capn3‐deficient mice in females (I). Regarding average speed, female Capn3‐deficient mice were faster at 6 weeks of age and slower at 12 weeks of age, compared to WT mice (J). Male Capn3‐deficient and WT mice did not present differences in maximum (K) or average speed (L). Asterisks indicate significant differences comparing genotypes within timepoints. Timepoints: t0 = 6 weeks, t1 = 12 weeks, t2 = 20 weeks = 5 months, t3 = 28 weeks, t4 = 36 weeks, t5 = 44 weeks, t6 = 52 weeks, t7 = 60 weeks, t8 = 68 weeks.

Mean duration (Figure 1A) and distance (Figure 1B) of a session differed significantly comparing female WT and Capn3‐deficient mice at timepoint 1 (12 weeks of age), with WT mice running a slightly shorter distance (distance WT 5679 ± 934.5 cm, Capn3‐deficient 7724 ± 1220 cm, *p* = 0.04) and for a shorter duration (duration WT 2.16 ± 0.39 min, Capn3‐deficient 3.14 ± 0.48 min, *p* = 0.02). Regarding males, mean duration (Figure 1C) and distance (Figure 1D) did not differ at any timepoint (*p* > 0.05). Comparing mean duration of a single run without interruption of running (Figure 1E), female Capn3‐deficient mice (0.52 ± 0.03 min) ran slightly shorter (*p* = 0.04) than WT mice (0.60 ± 0.05 min) at the age of 36 weeks (t4), while mean distance of a single run did not differ at any timepoint (Figure 1F, all *p* > 0.05). Interestingly, male WT and Capn3‐deficient mice did not present relevant differences regarding mean duration of a single run (Figure 1G, *p* > 0.05), but mean distance of a single run (Figure 1H) was slightly reduced in Capn3‐deficient mice from t3 until t5 (28–44 weeks of age) (t3: WT 483.1 ± 57.66 cm, Capn3‐deficient 368.1 ± 45.45 cm, *p* = 0.02; t4: WT 522.1 ± 72.86 cm, Capn3‐deficient 407.0 ± 33.00 cm, *p* = 0.04; t5: WT 599.0 ± 93.24 cm, Capn3‐deficient 451.0 ± 73.02 cm, *p* = 0.049). Maximum speed did not differ significantly between genotypes in female mice (Figure 1I). Average speed (Figure 1J) was slightly higher in female Capn3‐deficient mice compared to WT at the age of 6 weeks (t0: WT 0.25 ± 0.01 m/s, Capn3‐deficient 0.28 ± 0.01 m/s, *p* = 0.016) and slightly higher in WT mice at the age of 12 weeks (t1: WT 0.35 ± 0.01 m/s, Capn3‐deficient 0.30 ± 0.02 m/s, *p* = 0.03). In male mice, no differences ocurred in maximum (Figure 1K) and average speed (Figure 1L).

Taken together, the mean distance of a single run was reduced in male Capn3‐deficient mice for a time span of 4 months, until the age of about 10 months.

### 3.2 Quantitative MRI revealed significant genotype‐specific differences in wT2 relaxation time in gastrocnemius and soleus muscle at 15 months of age and differences in muscle composition related to sex and age were detected across all muscle groups

At 5 and 15 months of age, qMRI measurements of the left hindlimb were performed in three male and three female Capn3‐deficient and WT mice. At 15 months, lower leg muscles could be analyzed reliably; however, DTI analysis—particularly in female mice—was compromised by artifacts caused by impeded positioning of the mice, mainly due to the increased amount of surrounding adipose tissue.

**FIGURE 2:**
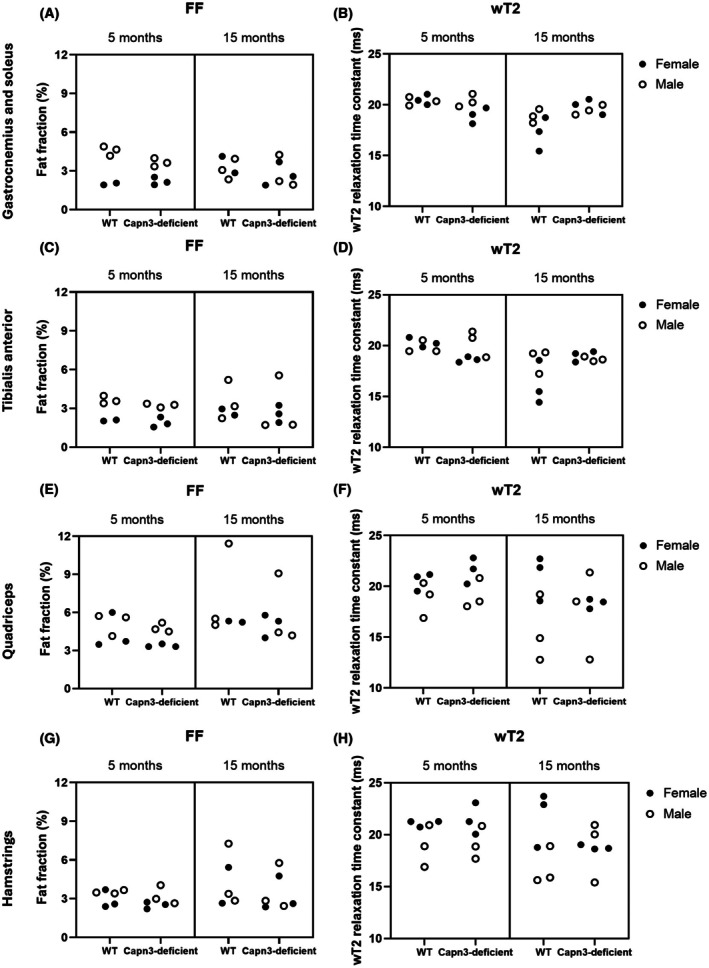
Results of MRI parameters fat fraction (FF) and water T2 relaxation time (wT2) in different muscle groups of male and female WT and Capn3‐deficient mice at 5 and 15 months of age are presented. In the calf (including gastrocnemius and soleus), significant effects were observed for sex and for the interaction between age and sex for the FF. Post hoc analysis for multiple comparisons revealed a significant higher FF in male WT mice compared to females at the age of 5 months (A). Regarding wT2, significant effects were found for age, genotype × age and genotype × age × sex. Post hoc analysis revealed a reduction in wT2 in female WT mice between 5 and 15 months of age. Additionally, wT2 was significantly higher in Capn3‐deficient females at 15 months of age compared to WT controls (B). In tibialis anterior muscle, results for FF showed a significant effect for sex; however, post hoc multiple comparisons did not reveal significant differences between individual groups (C). For wT2, significant effects were observed for age, genotype × age and genotype × age × sex. Post hoc analysis demonstrated a decrease in wT2 over time in female WT mice (D). In the upper leg, FF did not differ with respect to genotype, age, or sex in quadriceps muscles (E). In contrast, wT2 showed significant effects of sex in quadriceps muscles, without significant differences between individual groups in the post hoc analysis (F). In the hamstrings, FF did not differ with respect to genotype, age, or sex (G), while wT2 showed significant effects of sex, again without significant differences in the post hoc analysis (H).

In the calf (including gastrocnemius and soleus), significant effects were observed for sex (*F* (1, 14) = 8.648, *p* = 0.0107) and for the interaction between age and sex on FF (*F* (1, 14) = 11.57, *p* = 0.0043. See Figure 2A). Post hoc analysis for multiple comparisons revealed a significantly higher FF in male WT mice (4.57% ± 0.36%) compared to females (1.99% ± 0.1%) at the age of 5 months (*p* = 0.0493). Regarding wT2, significant effects were found for age (*F* (1, 16) = 12.47, *p* = 0.0028), for the interaction between genotype and age (*F* (1, 16) = 12.61, *p* = 0.0027) and the interaction between genotype, age and sex (*F* (1, 16) = 7.315, *p* = 0.0156. See Figure 2B). Post hoc analysis revealed a reduction in wT2 in female WT mice between 5 months (20.5 ± 0.5 ms) and 15 months of age (17.19 ± 1.7 ms; *p* = 0.0030). Additionally, wT2 was significantly higher in Capn3‐deficient females at 15 months of age (19.87 ± 0.77 ms) compared to WT controls of the same age (*p* = 0.0189).

In tibialis anterior muscle, results for FF showed a significant effect of sex (*F* (1, 14) = 5.122, *p* = 0.0400); however, post hoc multiple comparisons did not reveal significant differences between individual groups (Figure 2C). For wT2, significant effects were observed for age (*F* (1, 16) = 15.09, *p* = 0.0013), the interaction between genotype and age (*F* (1, 16) = 5.652, *p* = 0.0303) and the interaction between genotype, age and sex (*F* (1, 16) = 8.415, *p* = 0.0104). Post hoc analysis demonstrated a decrease in wT2 over time in female WT mice (5 months: 20.30 ± 0.48 ms; 15 months: 16.15 ± 2.14 ms; *p* = 0.0034. See Figure 2D).

In the upper leg, FF did not differ with respect for genotype, age, or sex in quadriceps or hamstring muscles. In contrast, wT2 showed significant effects of sex (quadriceps: *F* (1, 16) = 7.602, *p* = 0.0140; hamstrings: *F* (1, 16) = 9.617, *p* = 0.0069), again without significant differences between individual groups in the post hoc analysis (Figure 2E–H).

**FIGURE 3:**
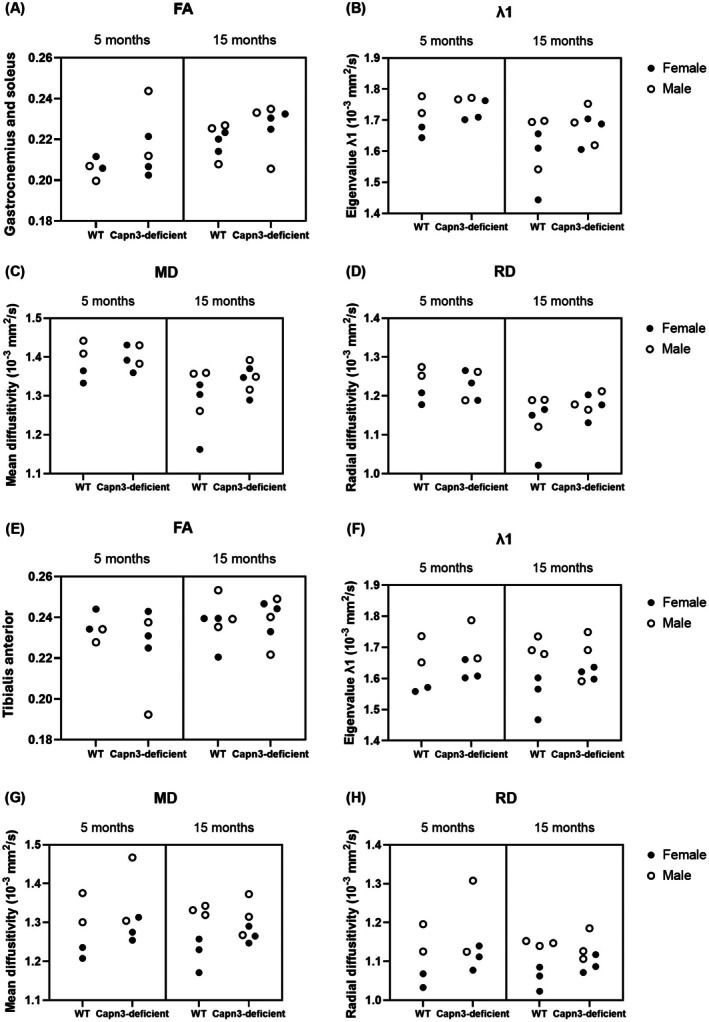
Results of diffusion parameters fractional anisotropy (FA), eigenvalue *λ*1 (*λ*1), mean diffusivity (MD) and radial diffusivity (RD) in muscles of the lower leg of male and female WT and Capn3‐deficient mice at 5 and 15 months of age are presented. Diffusion parameters in the calf muscles (gastrocnemius and soleus) showed significant effects for age for FA, *λ*1, MD and RD. However, post hoc multiple comparisons did not reveal significant differences between individual groups (A–D). In the tibialis anterior muscle, FA did not show significant differences (E), whereas *λ*1 (F), MD (G) and RD (H) exhibited significant effects of sex. Again, post hoc analysis did not demonstrate significant differences between individual groups.

Diffusion parameters in the calf muscles showed significant effects of age on FA (*F* (1, 13) = 4.797, *p* = 0.0473), *λ*1 (*F* (1, 13) = 7.784, *p* = 0.0153), MD (*F* (1, 13) = 10.77, *p* = 0.0060) and RD (*F* (1, 13) = 12.15, *p* = 0.0040). However, post hoc multiple comparisons did not reveal significant differences between individual groups (Figure 3A–D). In the tibialis anterior muscle, FA did not show significant differences, whereas *λ*1 (*F* (1, 13) = 21.38, *p* = 0.0005), MD (*F* (1, 13) = 20.98, *p* = 0.0005) and RD (*F* (1, 13) = 18.16, *p* = 0.0009) exhibited significant effects of sex. Again, post hoc analysis did not demonstrate significant differences between individual groups (Figure 3E–H).

In the upper leg (quadriceps and hamstrings), diffusion parameters, which were only analyzed for males and *n* = 1 female Capn3‐deficient mouse at 15 months of age (due to the abovementioned artifact issue), did not reveal significant differences between genotypes or sexes at 5 months of age. In male mice, no significant effects of age or genotype were detected in quadriceps muscle at 15 months of age.

In contrast, hamstring muscles showed a significant effect of age on MD (*F* (1, 6) = 27.18, *p* = 0.0020), with post hoc analysis revealing significantly higher MD values in male Capn3‐deficient mice at 5 months (1.46 ± 0.0008 10^−3^ mm^2^/s) compared to 15 months of age (1.33 ± 0.03 10^−3^ mm^2^/s; *p* = 0.0021). Similar findings were observed for RD with significant effects of age (*F* (1, 6) = 45.06, *p* = 0.0005). Post hoc analysis revealed significant differences (*p* = 0.0159) between male WT mice at 5 (1.25 ± 0.05 10^−3^ mm^2^/s) and 15 months of age (1.18 ± 0.01 10^−3^ mm^2^/s), as well as between male Capn3‐deficient mice at 5 (1.31 ± 0.0210^−3^ mm^2^/s) and 15 months (1.17 ± 0.02 10^−3^ mm^2^/s; *p* = 0.0008). See Figure S3 for details.

### 3.3 Detection of inflammatory infiltrates in the soleus muscle as a possible histological correlate for higher wT2 relaxation time in calf muscles of Capn3‐deficient mice

**FIGURE 4:**
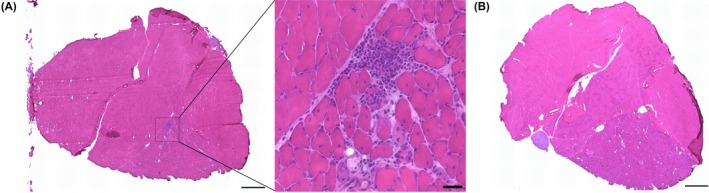
H&E staining of a cross‐section of gastrocnemius and soleus muscle of one Capn3‐deficient mouse at the age of 15 months (A, left) and enlargement of an endomysial inflammatory infiltrate in the soleus muscle (A, right). H&E staining of a cross‐section of gastrocnemius and soleus muscle of one WT mouse at the age of 15 months (B). Scale bar left: 500 μm, right 50 μm.

At the age of 15 months, significantly higher wT2 relaxation times were observed in gastrocnemius and soleus muscles of Capn3‐deficient mice compared to WT mice. To validate this finding histologically, we performed H&E staining of a cross‐sectional area of gastrocnemius and soleus muscles of Capn3‐deficient (Figure 4A) and WT mice (Figure 4B) at the age of 15 months. We detected endomysial inflammatory infiltrates in the soleus muscles of Capn3‐deficient mice, which may correspond to the higher wT2 relaxation time observed, a known marker for active muscle damage such as inflammation or myoedema.^13^

## 4 DISCUSSION

Recent work show that muscle qMRI is a sensitive, noninvasive biomarker across species, capable of detecting muscle fat replacement before clinical abnormalities become apparent in neuromuscular diseases such as Pompe disease or myotonic dystrophy type 1.^11^, ^22^, ^30^, ^31^ To assess its role in calpainopathy, we longitudinally analyzed qMRI in a mouse model known to develop only mild motor impairment.^23^

At 5 months, no genotype differences were detected, but by 15 months Capn3 deficiency caused modest yet significant wT2 elevations in the gastrocnemius and soleus muscles. Since wT2 enhancement has been proposed as a marker of active muscle damage in humans^13^ and mice,^32^ these findings likely reflect inflammation, which is supported by the endomysial immune cell infiltration we observed in muscle biopsies. In calpainopathy patients, eosinophil accumulation and Interleukin‐32 upregulation have been reported,^33^, ^34^ while qMRI showed increased wT2 in non‐fat‐replaced muscles.^10^ These findings support inflammation as a key factor in early disease stages. Longer follow‐up beyond 15 months could clarify whether imaging changes precede structural and functional decline.

Considering absolute wT2 values, the higher wT2 in 15‐month‐old female Capn3‐deficient mice compared to WT appears to be mainly due to a reduction of wT2 in wild‐type mice during aging rather than a true increase in mutants. Porcari and colleages^35^ found that water diffusivity decreases with age in mouse muscles, possibly due to reduced myofiber water content or altered sarcolemmal properties. Similarly, a prior 8‐months age‐span study^21^ showed a comparable reduction in wT2. Thus, the wT2 changes at 15 months in transgenic mice may indicate inflammation within the soleus muscle that offsets age‐related water loss. In contrast, in humans an increase of T2 was detected with age.^36^

Previous studies in various calpainopathy mouse models, including the one used in this study, identified the soleus, psoas, deltoid, and diaphragm as the most affected muscles, while tibialis anterior, gastrocnemius, and quadriceps are less impacted.^23^, ^37^, ^38^ Our qMRI protocol, adapted from a standard human neuromuscular imaging protocol,^39^ focused on hindlimb muscles. We segmented hamstrings, quadriceps, tibialis anterior, and gastrocnemius together with soleus for separate analysis, as these are easily distinguishable in mice.^21^

Although this protocol effectively detected diffusion changes in a mouse model of the metabolic myopathy Pompe disease, it may be less sensitive for calpainopathy, since the only severely affected muscle within the scanned region—the soleus—is analyzed together with the larger gastrocnemius. Thus, the setup might be more suitable for other myopathy models. Nonetheless, our protocol remains among the most accurate for differentiating mouse muscle by qMRI, compared with prior studies that analyzed entire muscle tissue,^31^, ^40^ single slices,^41^ or small regions of tibialis anterior and gastrocnemius.^35^

We did not observe significant differences in FF between WT and Capn3‐deficient mice at either 5 or 15 months of age. This suggests that the model represents an early disease stage, before muscle‐fat replacement occurs, which may also explain the absence of motor deficits.^23^ Alternatively, the lack of fat replacement could reflect a general feature of mouse models for neuromuscular diseases.^21^ Although diffusion parameters are considered early markers of muscle pathology, no changes were detected in this calpainopathy model. Typically, increased FA reflects fiber atrophy, and higher MD indicates edema, altered membrane permeability, or hypertrophy.^14^, ^15^ Given that histopathology revealed fiber atrophy and centralized nuclei within 15 months,^23^ we expected increased FA and reduced RD and MD in gastrocnemius and soleus. However, Rohm and colleagues^21^ showed that diffusion barriers within myofibers affect the diffusion signal more strongly than fiber size, due to short diffusion times used for acquisition. Consequently, the absence of diffusion changes may relate to insufficient diffusion time and the relatively high inter‐animal variability^21^ combined with the small sample size.

In this study, we included mice of both sexes and found several sex‐related differences independent of genotypes. At 5 months, males showed a significantly higher fat fraction in the lower leg and in general lower wT2 relaxation times in thigh muscles compared to females, even though the post hoc analysis did not reveal significant differences between individual groups. Some diffusion parameters also showed differences regarding sex and age. Sex‐related differences in muscle composition are well established in humans; for instance, males have fewer type I fibers and higher glycolytic activity in the vastus lateralis.^42^ Güttsches and colleagues^43^ demonstrated that diffusion parameters reflect muscle fiber composition changes, which are influenced by sex,^42^ aging,^44^ exercise,^45^ and neuromuscular disease.^46^ In mice, the tibialis anterior consists mainly of fast‐twitch fibers.^47^ Thus, the observed diffusion parameter variations likely reflect sex‐ and age‐related differences in muscle composition. Although the lack of histological data and the low number of mice included in this study limit interpretation, future studies combining qMRI with histology in both sexes are warranted. Our findings emphasize that sex differences may significantly influence muscle qMRI results, underscoring the importance of including both sexes in preclinical studies.

In addition to muscle qMRI, we evaluated whether automated running wheel analysis could reveal subtle motor impairments in the calpainopathy mouse model, which shows no abnormalities in standard motor tests, such as grip strength, four limb wire hanging test and beam walk test.^23^ No significant longitudinal differences were detected. At early timepoints, minor variations in running activity occurred between female WT and Capn3‐deficient mice, likely reflecting behavioral or environmental influences rather than genotype effects. In males, Capn3‐deficient mice showed a transient reduction in mean running distance between 6 and 10 months, followed by a decline in both genotypes at later timepoints. These findings may indicate mild alterations in motor activity among male mutants. Overall, however, no consistent motor phenotype was identified in Capn3‐deficient mice using the IPHS system.

The main limitation of this study is the small sample size (*n* = 4 per genotype and sex), which, due to sex stratification, increases interindividual variability and reduces statistical power. A potential bias in IPHS data may also have resulted from housing both genotypes together.^21^, ^48^ Moreover, mice were only observed until 15 months of age; since MRI differences were minor at this stage, extended observation at older ages would be valuable. During diffusion MRI of aging female mice, data quality was reduced, with motion or positioning artifacts affecting 5 of 6 animals at 15 months. This may relate to the fact that our diffusion protocol had not previously been applied to mice older than 8 months.^21^ Other qMRI protocols were only used for animals aged 2–5 months.^31^, ^40^, ^41^ Porcari and colleagues^35^ scanned 44‐week‐old mice without image issues but focused only on the lower leg, whereas our extended protocol performed well overall.

In conclusion, IPHS and muscle qMRI offer advanced and objective methods with a low burden for the mice for detecting subtle motor and structural abnormalities in mice compared to common tests for motor function. These techniques are particularly valuable for models without clear motor phenotypes, such as calpainopathy. Here, qMRI revealed significant wT2 increases in calf muscles of 15‐month‐old transgenic mice, consistent with endomysial inflammation, while IPHS suggested mildly reduced motor activity in male transgenics. Future work should include larger cohorts, separate housing of genotypes, and longer observation periods to improve statistical robustness and detect late‐onset pathology.

## AUTHOR CONTRIBUTIONS

**Nicolina Südkamp:** Conceptualization; data curation; formal analysis; funding acquisition; investigation; methodology; project administration; validation; visualization; writing – original draft; writing – review and editing. **Marlena Rohm:** Data curation; formal analysis; investigation; methodology; software; supervision; writing – review and editing. **Gabriele Russo:** Data curation; investigation; writing – review and editing. **Xavier Helluy:** Methodology; writing – review and editing. **Abdulhadi Kocabas:** Formal analysis; software; writing – review and editing. **Martijn Froeling:** Formal analysis; methodology; software; writing – review and editing. **Felix Kleefeld:** Writing – review and editing. **Denise Manahan‐Vaughan:** Resources; writing – review and editing. **Tobias Ruck:** Writing – review and editing. **Frank Jacobsen:** Data curation; investigation; methodology; writing – review and editing. **Matthias Vorgerd:** Conceptualization; funding acquisition; methodology; project administration; resources; supervision; validation; writing – review and editing. **Johannes Forsting:** Formal analysis; software; visualization; writing – review and editing. **Lara Schlaffke:** Conceptualization; formal analysis; funding acquisition; methodology; project administration; software; supervision; validation; writing – original draft; writing – review and editing.

## FUNDING INFORMATION

Open Access funding was enabled and organized by DEAL Consortium (https://deal‐konsortium.de). N.S. received funding from the FoRUM‐program of the Ruhr‐University Bochum (K154‐21). We highly appreciate funding from the Heimer Foundation. D.M.‐V. received funding from InnovationsForum No. IF031‐22 from Ruhr‐University Bochum.

## CONFLICT OF INTEREST STATEMENT

The authors declare no competing interests.

## ETHICS STATEMENT

All animal experiments were performed following the German Law on the protection of animals (TierSchG §§ 7–9) and were approved by the local ethics committee of the state authority (LAVE, North‐Rhine‐Westphalia, Germany, 81‐02.04.2021.A444).

## Supporting information


Data S1.

## Data Availability

Data and further information are available upon reasonable request made to nicolina.suedkamp@rub.de.
