# Supplementary material for: Evaluation of quantitative muscle MRI and an intelligent phenotyping housing system as advanced phenotyping methods in a mouse model of calpain 3‐deficient muscular dystrophy
Source: Animal Model Exp Med. 2026 Apr 6;9(7):1469–79. doi: 10.1002/ame2.70193 (PMC13394435; doi:10.1002/ame2.70193)
Supplement: Supplementary file 1 — Data S1. [file AME2-9-1469-s001.pdf]

Supplementary Information

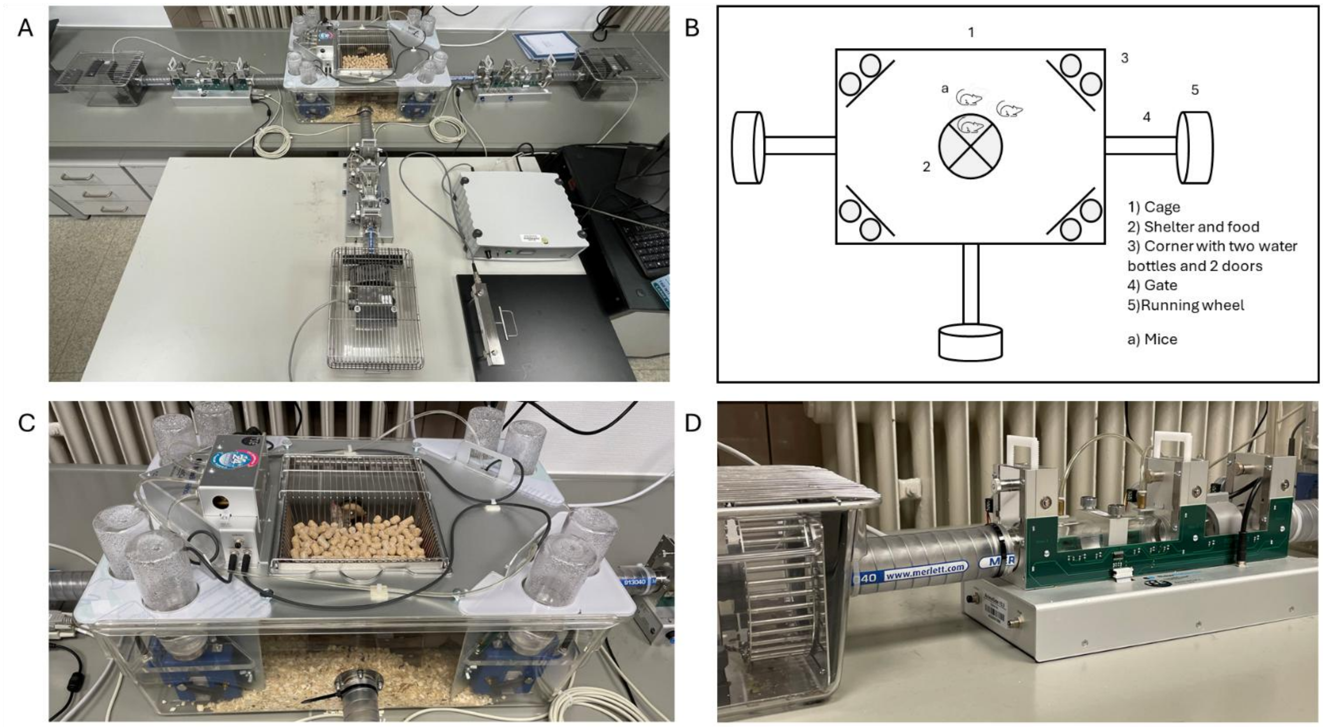

Figure S1

Whole IPHS set-up with cage and the running wheels (A), schematic set-up of the IPHS (B), cage (C) and gate with running wheel (D).

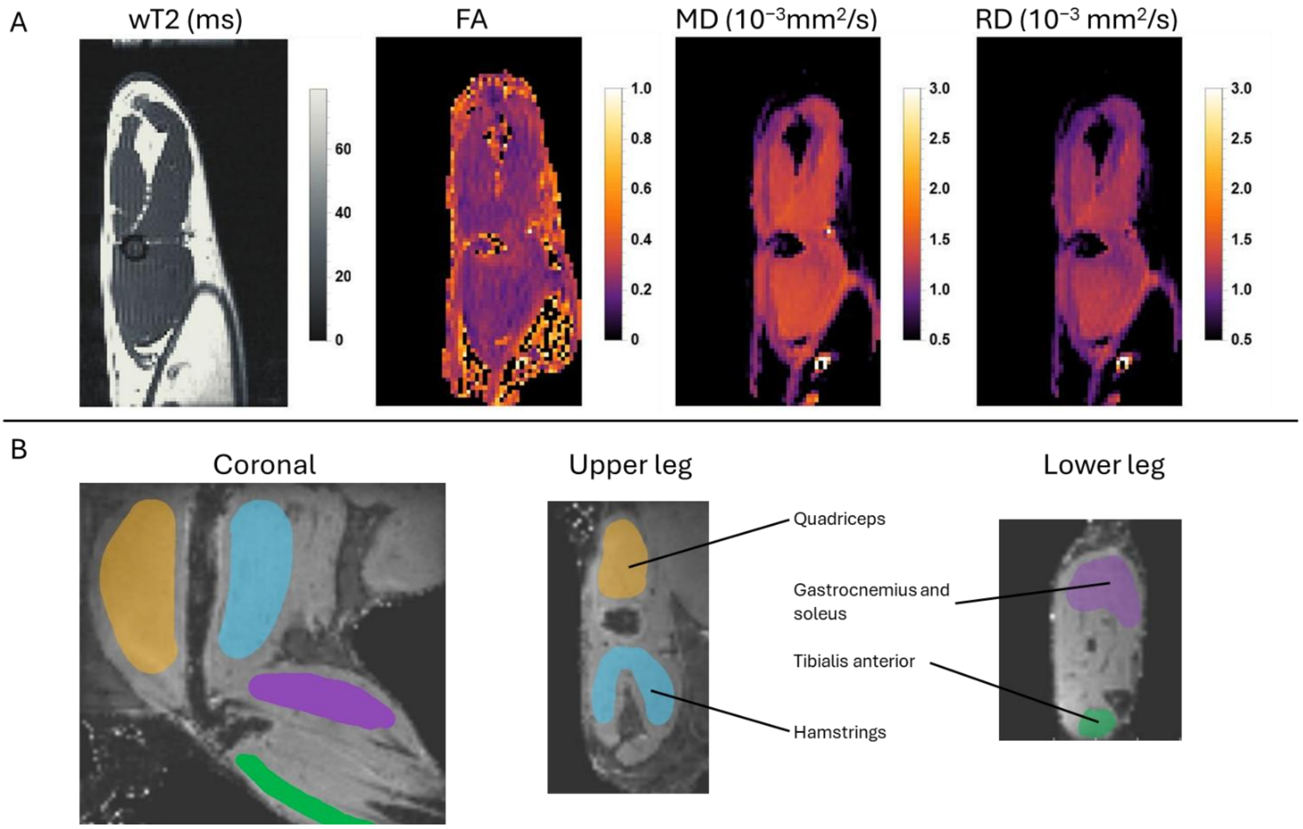

Figure S2

One example of processed data for a mouse at the age of 5 months (A), from left to right: water T2 (wT2), fractional anisotropy (FA), mean diffusivity (MD), radial diffusivity (RD). Example of manual segmentation with underlying outphase image (B): coronal cut to visualize the placement of the mice with upper and lower leg segmentations, labels are as indicated.

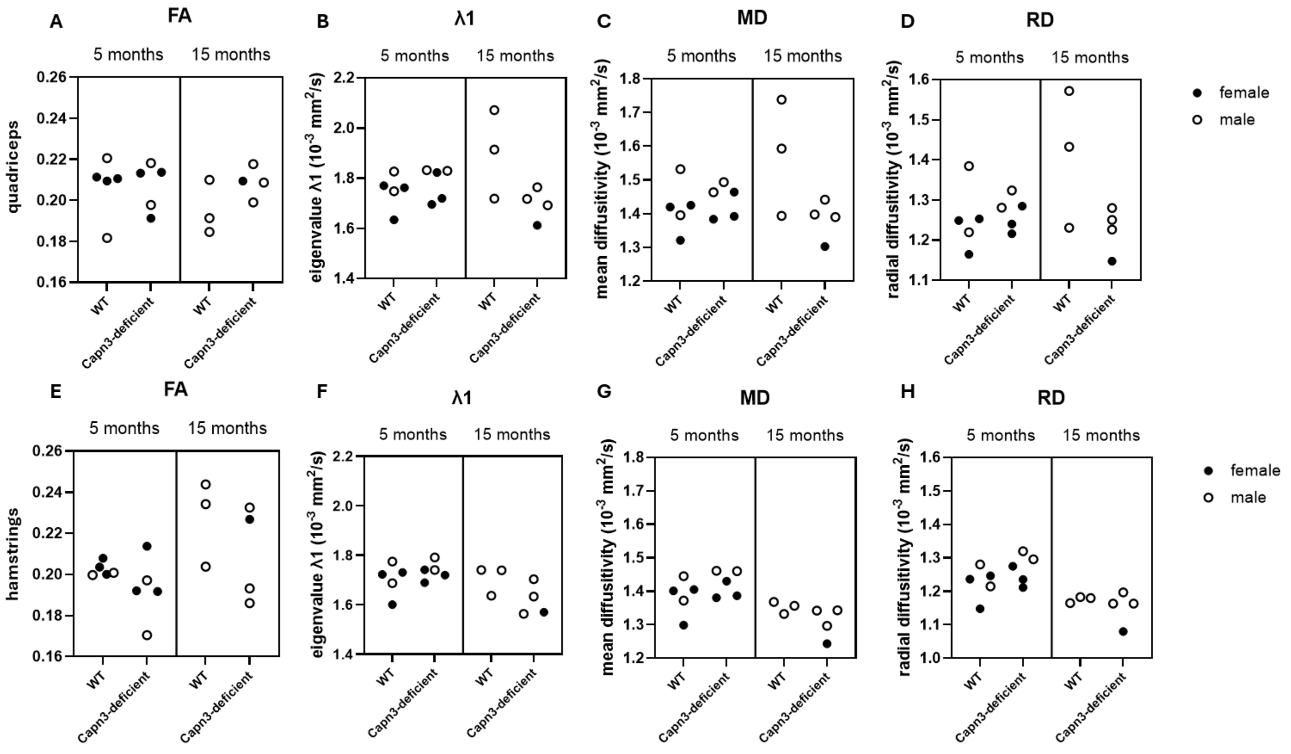

Figure S3

Results of diffusion parameters fractional anisotropy (FA), eigenvalue λ1 (λ1), mean diffusivity (MD) and radial diffusivity (RD) in muscles of the upper leg of male and female WT and Capn3-deficient mice at 5 and 15 months of age are presented. In the upper leg (quadriceps and hamstrings), diffusion parameters, which were only analyzed for males and n=1 female Capn3-deficient mouse at 15 months of age (due to artefact issue), did not reveal significant differences between genotypes or sexes at 5 months of age. In male mice, no significant effects of age or genotype were detected in quadriceps muscle at 15 months of age (A-D). Hamstring muscles also showed no significant effects of age or genotype on FA (E) and λ1 but showed a significant effect of age on MD (G), with post hoc analysis revealing significantly higher MD values in male Capn3-deficient mice at 5 months compared to 15 months of age. Similar findings were observed for RD(H) with significant effects of age. Post hoc analysis revealed significant differences between male WT mice at 5 and 15 months of age, as well as between male Capn3-deficient mice at 5 and 15 months.

Table S1

Scan parameters for water T2, diffusion-weighted imaging (DWI) and Dixon sequences. Abbreviations: multi-slice, multi-echo (MSME), multi-echo gradient echo (MEGE), field of view (FOV), repetition time (TR), echo time (TE)

	Water T2	***DWI***	***Dixon***
Sequence	**MSME**	**single-shot EPI**	**MEGE**
FOV (mm³)	18.3 x 15.6 x 5.25	18 x 10 x 5.25	20 × 15.6 × 20
Voxel size (mm³)	0.15 x 0.15 x 0.75	0.2 x 0.2 x 0.75	0.2 x 0.2 x 0.2
Slice Gap (mm)	0.0	-	-
Slices	7	7	50
TR (ms)	2200	2000	200
TE (ms)	4.7-84.9	18.06	1.9-19
Echo spacing (ms)	4.71	-	1.904
Number of echoes	18	-	10
b-values (number of gradients, mm²/s)	0	0 (n = 43), 10 (n = 18), 25 (n = 18), 100 (n = 18), 200 n = 36), 400 (n = 48), 600 (n = 72) Total number of gradient orientations: 253	0
Diffusion time	-	δDuration 3 ms ΔSeparation 10.04 ms	-
Fat Suppression	-	CHESS (Fat-Sat)	-
